# Broadband chirality-coded meta-aperture for photon-spin resolving

**DOI:** 10.1038/ncomms10051

**Published:** 2015-12-02

**Authors:** Luping Du, Shan Shan Kou, Eugeniu Balaur, Jasper J. Cadusch, Ann Roberts, Brian Abbey, Xiao-Cong Yuan, Dingyuan Tang, Jiao Lin

**Affiliations:** 1 Nanophotonics Research Centre, Shenzhen University & Key Laboratory of Optoelectronic Devices and Systems of Ministry of Education and Guangdong Province, College of Optoelectronic Engineering, Shenzhen University, Shenzhen 518060, China; 2 School of Electrical and Electronic Engineering, Nanyang Technological University, Singapore 639798, Singapore; 3 School of Physics, The University of Melbourne, Tin Alley, Melbourne, Victoria 3010, Australia; 4 Department of Chemistry and Physics, La Trobe Institute for Molecular Science (LIMS), La Trobe University, Melbourne, Victoria 3086, Australia; 5 Australian Research Council Centre of Excellence for Advanced Molecular Imaging, Australia; 6 School of Electrical and Computer Engineering, RMIT University, Melbourne, Victoria 3001, Australia

## Abstract

The behaviour of light transmitted through an individual subwavelength aperture becomes counterintuitive in the presence of surrounding ‘decoration', a phenomenon known as the extraordinary optical transmission. Despite being polarization-sensitive, such an individual nano-aperture, however, often cannot differentiate between the two distinct spin-states of photons because of the loss of photon information on light-aperture interaction. This creates a ‘blind-spot' for the aperture with respect to the helicity of chiral light. Here we report the development of a subwavelength aperture embedded with metasurfaces dubbed a ‘meta-aperture', which breaks this spin degeneracy. By exploiting the phase-shaping capabilities of metasurfaces, we are able to create specific meta-apertures in which the pair of circularly polarized light spin-states produces opposite transmission spectra over a broad spectral range. The concept incorporating metasurfaces with nano-apertures provides a venue for exploring new physics on spin-aperture interaction and potentially has a broad range of applications in spin-optoelectronics and chiral sensing.

## Abstract (web summary)

Nano-apertures cannot distinguish between distinct spin-states of photons because of information loss upon light-aperture interaction. Here, Du *et al.* report a subwavelength aperture integrated with metasurfaces which breaks spin degeneracy and produces opposite transmission spectra over a broad spectral range.

## Graphical abstract

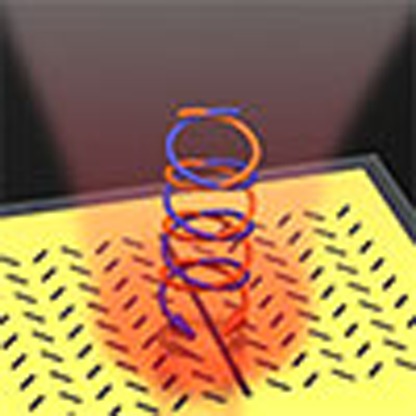

The optical response of an aperture perforating a smooth opaque film of, for example, metal, has been studied for decades. Early research into transmission through holes concentrated on isolated apertures for use at longer wavelengths^1^. Interest in the topic was revived by the observation of the phenomenon dubbed extraordinary optical transmission (EOT). This occurs when a subwavelength aperture appears in a periodic arrangement or with surrounding nanostructures^2^,^3^,^4^ and transmits significantly more light than in isolation. Typically these ‘decorations' include the 1D/2D assembly of homogenous apertures or patterning with nearby grooves, through which one can control the nature of the transmitted light^5^,^6^ such as enhancing^7^ or suppressing^8^ the total power transmitted, and ‘beaming'^9^ beyond the predictions of diffraction theory for an isolated hole.

The introduction of additional surface features enhances the light-aperture interaction via the excitation of electromagnetic surface waves^10^ (primarily surface plasmons (SPs), the collective charge density waves propagating on a smooth metal surface driven by optical radiation^11^), which bear the energies from incident light tuning the transmission properties of the optical aperture. The utilization of the arrangement of optical apertures, however, faces a couple of fundamental limitations. First of all, previous attention has concentrated on modulating the magnitude rather than the phase of surface waves emanating from surface features. Phase, however, plays a much more significant role in the transmission process involving complex near-field interference effects. Second, polarization has been often overlooked previously as unpolarized broadband sources are commonly used in experimental investigations. The optical transmission of subwavelength apertures is, however, extremely sensitive to the polarization state. For instance, a circular nanohole responds to the field components polarized perpendicular to its edge while being relatively insensitive to the parallel component. While the partial loss of this information from the incident beam will not cause the EOT process to disappear entirely, it does render the aperture unable to distinguish the chirality of a rotational polarization, for example, the spin state of circularly polarized light (CPL), which is an important degree-of-freedom of photons that is fundamental to many developments in modern physics^12^,^13^,^14^,^15^,^16^,^17^,^18^ and applications in, for example, communication^19^ and chiral sensing^20^.

Metasurfaces^21^, a surface assembly of nano-antennas or holes, offer a path to addressing the issues mentioned above. As a 2D planar variation on the concept of metamaterials, metasurfaces have rapidly gained profile in recent years owing to their flexibility and effectiveness in shaping the wavefront (phase) of a free space or surface wave^14^,^22^,^23^,^24^ while requiring a much simpler fabrication process compared with bulk structures. By integrating an optical aperture with metasurfaces, one can design ‘meta-apertures' that respond to all the degrees-of-freedom of incident light (frequency, amplitude, phase and polarization), providing us with more opportunities to control the properties of the transmitted light.

Here we demonstrate a meta-aperture that spectrally breaks the spin degeneracy of optical transmission through a subwavelength aperture. The introduced metasurfaces link the chirality of light with the aperture via SPs, evoking new physics related to the chirality-directed EOT process. Owing to the small dimensions of the resulting structure, the meta-apertures are well-suited to miniaturization and on-chip spin optoelectronic applications.

## Results

### Design of meta-aperture

**Figure 1:**
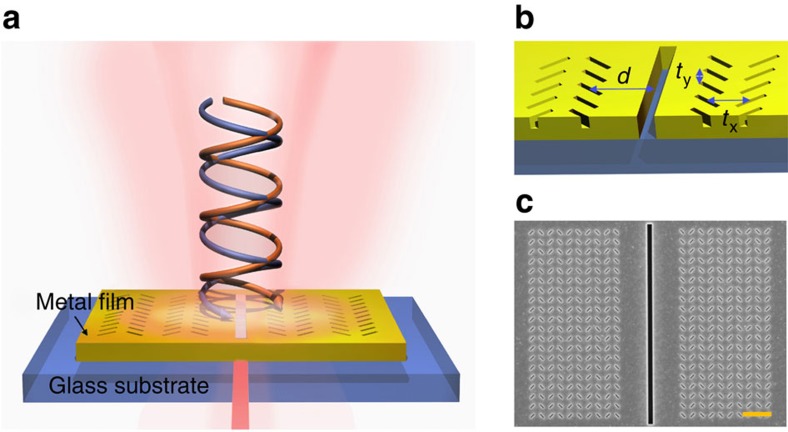
Design of a subwavelength meta-aperture which exhibits spin-resolved optical transmission. (**a**) Schematic diagram illustrates the composite of a typical meta-aperture and the illumination configuration. The meta-aperture is composed of an optically thick metal slit decorated with a pair of metasurfaces with 180° rotational symmetry. The metasurfaces are made up of multiple arrays of rectangular nanoholes with alternating orientations. (**b**) A cross-sectional schematic representation of the structure. (**c**) An scanning electron microscopy micrograph of an as-prepared meta-aperture in which 10 nanohole arrays are patterned on either side of the central slit. The scale bar, 1 μm.

A schematic illustration of the meta-aperture investigated in this article is shown in Fig. 1a. A pair of metasurfaces with 180° rotational symmetry is patterned on either side of a subwavelength metal slit. The metasurfaces are composed of multiple arrays of nanoholes (rectangular shape, without penetration through the film, see Fig. 1b), with lateral displacement equal to *t*_*x*_. The rectangular antennas in each column are orthogonal to the elements in the adjacent column (±*π*/4 with respect to the horizontal axis). The vertical displacement of the antennas within one column is denoted as *t*_*y*_. Figure 1c shows a scanning electron microscopy image of an as-prepared meta-aperture fabricated using a focused ion beam lithographic system on a 300-nm-thick gold film, which is deposited on a silica substrate. The central slit has a width of 200 nm. The dimension of each individual antenna is 200 × 50 nm, etched to a depth of 200 nm and separated by 330 nm in both the lateral and vertical directions (*t*_*x*_=*t*_*y*_=330 nm). Ten antenna columns are patterned on either side. The distance to the centre of the first column of antennas on each side is designated *d*.

### Transmission properties of meta-apertures

**Figure 2:**
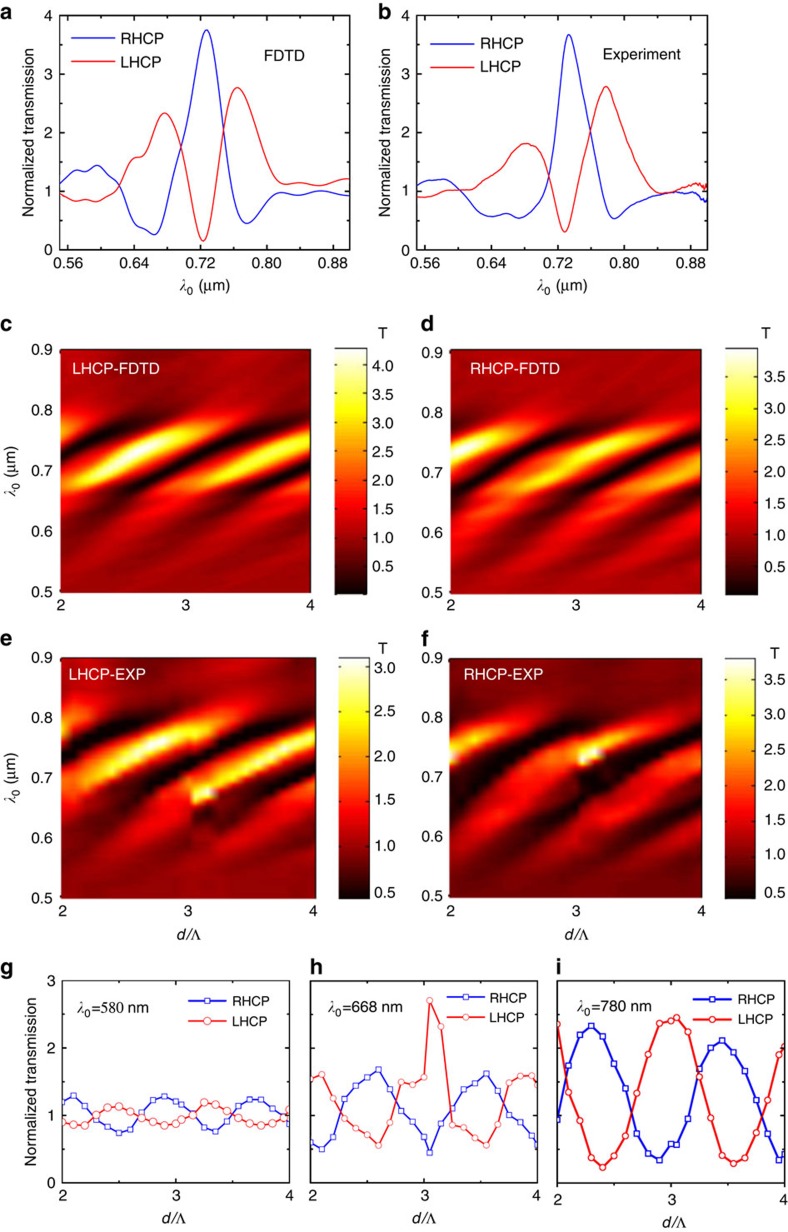
Optical transmission properties of the meta-apertures. (**a**) Simulated transmission spectra of the meta-aperture for different states of circularly polarized light. The transmission coefficient is normalized to that through an aperture-only configuration, illustrating the enhancement/depression of the transmittance modulated by the metasurfaces. The helicity-dependence of the meta-aperture is clearly seen. (**b**) Measured transmission spectra for both the left- and right-handed CPLs, verifying the numerical result. (**c**–**f**) 2D mapping of the optical transmissions obtained by changing the metasurface-to-slit distance. Top panels are the modelling results with FDTD and bottom panels the experimental ones. (**g**–**i**) Distance-dependent optical transmission at incident wavelengths of 580, 668 and 780 nm, respectively, obtained from **e**,**f**. These graphs illustrate the wavelength-dependent repetition of the transmission spectra linked to the dispersion relationship of SPs. The abnormal data at *d*=3Λ in **h** may be caused by a fabrication artefact, which supports localized plasmon mode and hence alters the transmission properties of the meta-aperture near the resonant wavelength.

The spin-dependent optical transmission spectra through the meta-apertures are shown in Fig. 2. Both the numerically modelled result calculated using a finite-difference time-domain (FDTD) algorithm (Fig. 2a) and the corresponding experimentally measured data (Fig. 2b) are shown. In the experiment, broadband light from a supercontinuum light source (NKT SuperK) is circularly polarized and slightly focused via a low numerical aperture (NA) objective lens (Olympus, × 4, NA=0.13); the illumination direction is normal to the fabricated structure. A collection objective lens (Olympus, × 60, NA=0.7) mounted on the reverse side of the meta-aperture is employed to collect the transmitted light through the slit, which is then directed into a spectrometer (OceanOptics, HR2000) for analysis. The transmission intensity is normalized to the transmission through an aperture-only configuration (denoted as *T*_norm_) to reveal the modulation effect of the metasurfaces on the transmission properties (Supplementary Fig. 1).

For each of the circular polarization states, the transmission spectra indicate an alternate enhancement (*T*_norm_>1) and depression (*T*_norm_<1) of the transmittance with respect to the slit in the absence of the metasurfaces. A remarkable phenomenon one can deduce from the spectra is the inverse response of the meta-aperture to the incident light helicity. The local maximum (minimum) in transmission for one handedness of CPL corresponds to the local minimum (maximum) in transmission for the other. The experimentally measured spectra in Fig. 2b reproduce well the numerical predictions except for a small global red-shift. To explore the influence of the slit-to-metasurface distances (denoted as *d*, see Fig. 1b), we fabricated additional metastructures of varying geometrical configuration. The simulation results for these structures are shown in Fig. 2c,d, while the corresponding experimental transmission spectra are shown in Fig. 2e,f. The results show that the spectral responses of the meta-apertures can easily be tuned by changing the groove-to-slit distance, with an increase in the distance causing a global red-shift of the transmission curves. At a fixed incident wavelength, the peaks/valleys of the spectra repeat at a pitch of *λ*_sp_, over which the SPs travel exactly one cycle at this wavelength. As a result, we can find a wavelength-dependent repetition rate of the transmission spectra (Fig. 2g-i), which is related to the dispersion relationship of SPs. The inverse EOT response of the meta-apertures to the spin state of the incident light persists for all of the distances considered, which is verified both by experiment and simulation.

### Interpretation of the spin-resolved EOT

**Figure 3:**
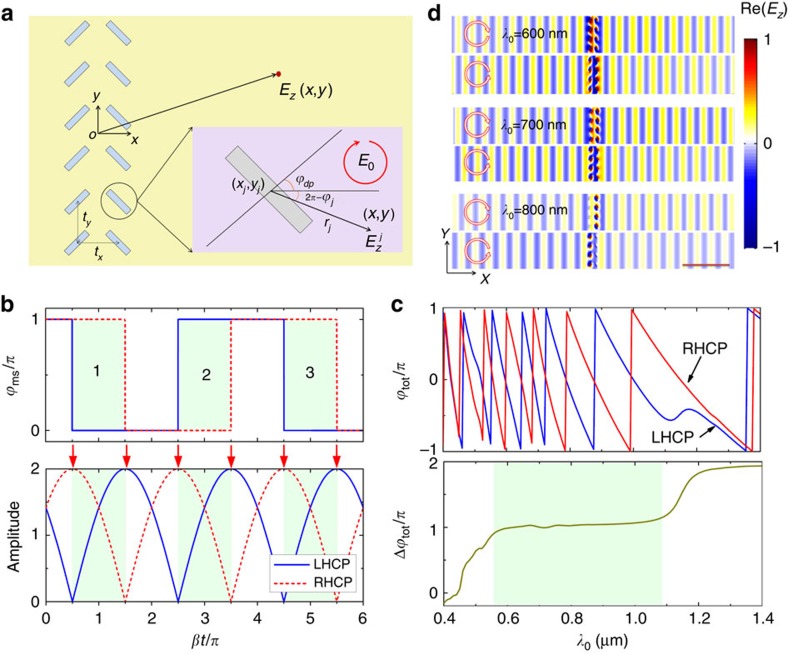
Tailoring the phase of surface plasmons via a metasurface. (**a**) Calculating the electric field of SPs (*E*_z_(*x*,*y*)) at an arbitrary point just above the metal surface emerging from a metasurface composed of a pair of antenna columns. (**b**) Analytically calculated phase component of SPs induced by the metasurface (*ϕ*_ms_) depicted in *k*-space (top panel), together with the amplitude of SPs modulated with the metasurface (bottom panel). One can see a pair of binary phases corresponding to the helicity pair of CPLs, with half-cycle shift in *k*-space. Such a half-cycle shift gives rise to multiple regions with *π*-phase difference (out-of-phase) between the left-and right-handed circularly polarized states. (**c**) Total phase (*ϕ*_tot_(*x*=*d*)) of SPs when travelling to the central slit obtained with our FDTD algorithm, confirming the *π*-phase difference. (**d**) The snapshots of the near-field SPs (Re(*E*_*z*_)) emerging from the metasurface at incident wavelengths of 600 nm, 700 nm and 800 nm, respectively. The metasurface couples the two circularly polarized free space waves into plasmon plane waves that are *π* out-of-phase. *t*_*x*_ and *t*_*y*_ are set to be 360 nm in all of calculations and simulations. The scale bar in **d** represents 3 μm.

Two mechanisms are involved in the EOT process: quasi-cylindrical waves (CWs), active only when the metasurface structure is very close to the aperture, and SP waves (dominant when *d* is large)^10^. In our designs, the metasurfaces are typically >2*λ*_sp_ distance from the central slit, leading to a relatively minor contribution of CWs to the transmission. To understand the physics behind the spin-resolved EOT, we performed an analytical study of SPs emitted from the metasurfaces. For simplicity, we consider initially the metasurface composed of a single pair of antenna columns situated to the left of the aperture (Fig. 3a). Each of the nano-antennas acts as an in-plane dipolar plasmon source, with dipole polarization perpendicular to its major axis, when CPL illuminates the metasurface at normal incidence. The superposition of the SP wavelets from an entire column of nano-antennas gives rise to an SP plane wave propagating perpendicular to and away from the column, as predicted by the stationary phase approximation (Supplementary Note 1).

The total intensity of SPs (where we consider only the right half space where the central slit is located) from the metasurface after the interference between the plasmon plane waves emitted from the two columns can, therefore, be mathematically expressed as:


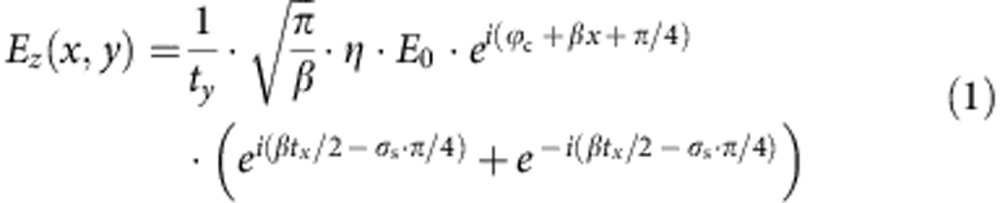


In equation (1), *E*_0_ denotes the amplitude of incident field, *η* the light-to-SP coupling efficiency, *ϕ*_c_ the coupling phase, *β* the wave-vector of the SPs and *σ*_s_ the spin state of incident light (+1 for a left-handed CPL (LHCP) and −1 for a right-handed one (RHCP)). We calculate only the z-component of SPs—the dominant field component determining the near-field properties of SPs. The terms within the bracket show the differential response of the metasurface to the incident light helicity. Their consolidated phase is denoted as *ϕ*_ms_, representing the phase component of SPs induced by the metasurface:


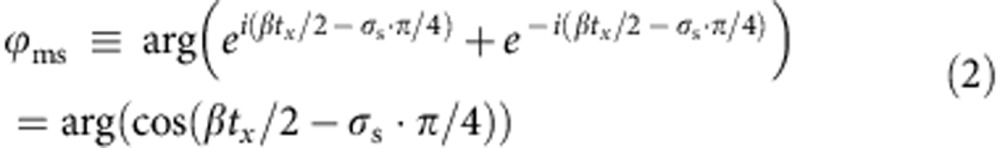


Interestingly, for each helicity, the two antenna columns yield conjugated phase terms. This means that the consolidated phase is determined solely by the sign of an oscillation function. This leads to a plasmonic binary phase repeating at ‘0' and ‘*π*' (Fig. 3b, top panel). The helicity of photons (*σ*_s_), appearing in the oscillation function, therefore, leads to a half-cycle shift between the binary phases from the RHCP and LHCP. Such a half-cycle shift gives rise to multiple wave-vector regions with *π* phase difference (green areas). In the analytical model of equation (1), we do not consider the scattering loss of the SP wave when it propagates through the adjacent antenna array. Such a treatment simplifies the analysis and gives us a clearer picture of the essential physics behind the spin-resolved EOT without losing much accuracy. (The influence of such scattering effect on SPs can be found in Supplementary Fig. 2).

Assuming the slit is located at *x*=*d*, the total accumulated phase of SPs when travelling to the slit is *ϕ*_tot_(*x*=*d*)=*π*/4+*ϕ*_c_(*λ*_0_)+*βd*+*ϕ*_ms_, including the coupling phase (*ϕ*_c_), the propagating phase (*βd*), the binary phase induced by the metasurface (*ϕ*_ms_) plus a constant phase term. The total phase as a function of wavelength is shown in Fig. 3c, obtained numerically via FDTD calculations. The broadband SP *π*-phase difference between circular polarization states can clearly be seen in Fig. 3c and be visualized in the near-field SP distributions for incident wavelengths of 600, 700 and 800 nm, shown in Fig. 3d.

Based on the near-field interference model^6^, the SPs are coupled back to free space radiation when they encounter the slit, and interfere with the photons incident directly on the aperture. Their constructive/destructive interference leads to an enhancement/reduction in the optical transmission through the aperture. As a result, the peak transmission (in-phase with the incident photons) for one handedness will coincide with a minimum in transmission (out-of-phase) for another handedness, due to the helicity response of the meta-aperture. The total accumulated phase, on the other hand, is modulated with the propagating phase (*βd*) that monotonically increases when decreasing the wavelength (Fig. 3c, top panel). This leads to an alternate peak/trough in transmission through the aperture as illustrated in Fig. 2. The small global red-shift of the experimental spectra with respect to those simulated (Fig. 2a,b) can be attributed to the deviation of the fabricated rectangular nanoholes from those designed in terms of shape, size and depth, which will alter the coupling phase (*ϕ*_c_) of SPs from free space light^25^,^26^ and the differences in optical constants of bulk material (used in FDTD) and actual thin films.

It is worth mentioning that, at the end points of the out-of-phase regions, that is, *βt*=(2*n*+1)˙*π*/2, *n*=0,1,2,…., (indicated with red arrows in Fig. 3b) the SPs exhibit spin-dependent unidirectional emission, as investigated in ref. 14. This type of amplitude modulation of SPs exists only at a set of discrete wavelengths, in contrast to the meta-aperture making use of the phase modulation that exhibits broadband features. Furthermore, if we integrate the same aperture with the metasurfaces of ref. 14, it will, to some extent, yield helicity-dependent optical transmission owing to the planar chirality of the structure (Supplementary Fig. 3). Nevertheless, it does not yield a chiral-EOT with opposite spin response. The metasurfaces should be carefully designed to encode the photon chirality information into the phases of SPs, or other types of surface waves, as demonstrated in this work.

## Discussion

**Figure 4:**
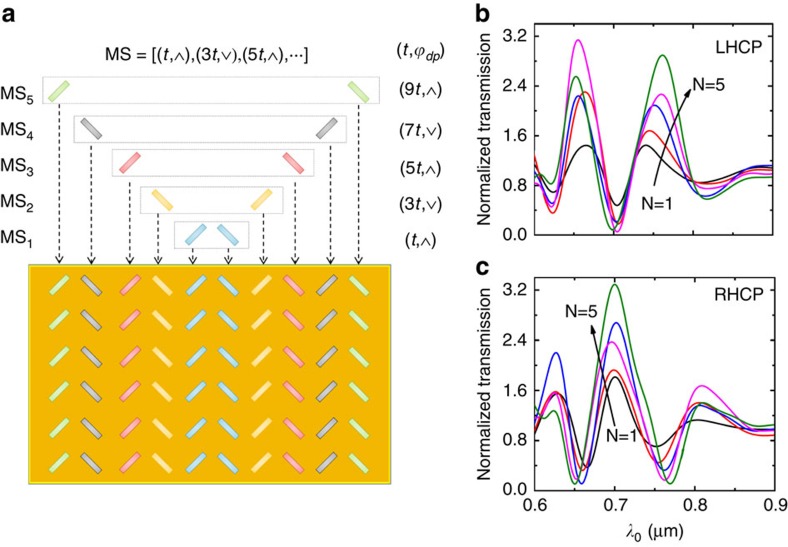
Enhancing the extinction ratio of the optical transmission by increasing the number of metasurface seeds. (**a**) Schematic illustration of the process for enhancing the extinction ratio with multiple metasurface seeds. The final metasurface is viewed as an assembly of five metasurface seeds with alternate orientation and 2*t* increment of the separation, which provided the base seeds with a separation of *t*. (**b**,**c**) The simulated transmission spectra through various meta-apertures with increasing number of seeds, under the illumination with left-handed and right-handed CPL, respectively.

In practice, we need to increase the number of antenna arrays beyond the pair of columns discussed above, to enhance the intensity of the SPs to improve the extinction ratio, as was done in our experiments (Fig. 1c). The basic principle of the process is that the out-of-phase feature of the structure should not be modified when increasing the number of nanoantenna column arrays. One may easily regard the left-side pattern in Fig. 1c as a simple periodic extension of the structure in Fig. 3a. This then appears to be counterintuitive since a periodic structure typically should not present a broadband feature. Figure 4a provides a schematic for interpreting the metasurface fabricated in our experiment. To clearly convey the concept, a pair of antenna arrays as shown in Fig. 3a is termed a ‘metasurface seed', which possesses a plasmonic binary phase BP_*σ*s_(*t*, *ϕ*_dp_) that is determined by the separation (*t*_*x*_=*t*_*y*_≡*t*) and orientation (*ϕ*_dp_) of the structure, where *σ*_s_ represents the spin state of incident photons (*σ*_+_ for LHCP and *σ*_−_ for RHCP). The entire structure is considered to be an assembly of multiple seeds with alternate orientation and a 2*t* increment in separation. Since all of the seeds share the same geometric centre, the consolidated phase of the metasurface is simply the summation of all of the plasmonic binary phases from the seeds:


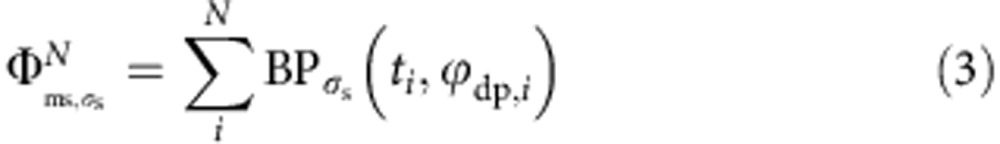


Mathematically, the out-of-phase/in-phase feature of the metasurface responding to the spin pair of CPL can be characterized by multiplying the consolidated phases:


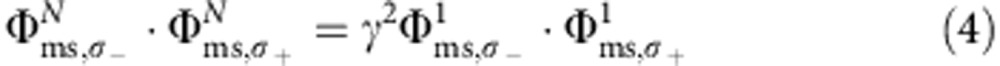


where *γ* is a coefficient related to the number of seeds (Supplementary Note 2). It is found that the process of increasing the number of antenna arrays maintains the relative phases between SP waves from LHCP and RHCP (Supplementary Fig. 4). Figure 4b,c shows the optical transmission spectra through a set of meta-apertures with increasing number of seeds, with the process as described. The distance between the geometric centre and the slit is fixed for all of the meta-apertures. As predicted, the shape of the transmission curve is maintained during the process while the extinction ratio is greatly improved owing to the enhanced intensity of SP waves.

Practically, such kind of meta-apertures can be coated on to the window of a photodiode, as a miniaturized CPL analyzer for resolving the photon spins (Supplementary Fig. 5). The performance of spin-photodiode (in terms of extinction ratio) can be improved by optimizing the structural parameters of the meta-aperture to match the amplitude of SPs with the directly transmitted wave to yield a completely destructive interference for a particular wavelength. In contrast to the CPL analyzer utilizing 3D/planar metamaterials^27^,^28^, our design simplifies the fabrication process and extends the operating wavelength from the near/mid-infrared to the visible region. In addition, the far-field detection scheme exhibits many advantages over near-field approaches that require a near-field probe (for example, near-field scanning microscope^14^,^29^ or fluorescent molecules^30^) to read out the spin information, greatly complicating the process and impairing the performance. The proposed meta-aperture could find valuable applications in bio-molecule and drug analysis (measuring the circular dichroism to determine the stereochemistry of target molecules) and creates a route to on-chip spin optoelectronic devices.

## Methods

### Numerical simulation

All of the numerical simulation work was performed with the commercial software FDTD Solutions from Lumerical Solutions, Inc. In the simulation, the refractive index of silica substrate was set to be 1.51; the dielectric constant of gold film was from the build-in material database within the software, in which the raw data is from ref. 31. Perfectly matched layer boundaries were employed for the *X* and *Z* directions while for the *Y* direction the boundary condition was set to be periodic to save the computation time. Such treatment will not cause significant deviation with the experimental model provided the repetition of nano-grooves at *Y* direction is large enough (20 times in the experiment, as can be seen in Fig. 1c). A global mesh size of 40 nm was applied in the calculation, while a finer mesh size of (2, 2, 10 nm) was employed for the fine structure of metasurface and (20, 2, 10 nm) for the slit.

### Experimental measurement

The experiments were carried out on a home-built microscopic system (Supplementary Fig. 1). An NKT supercontinuum source is illuminated normally on to the meta-aperture via a low NA objective lens (Olympus, × 4, NA=0.13). A combination of linear polarizer and quarter waveplate was employed to shift the incident polarization between left-handed and right-handed circular polarization. The transmitted light through the meta-aperture is collected with another objective lens ( × 60, NA=0.7), and is subsequently directed respectively to a CCD camera and a spectrometer with a cubic beam splitter. The CCD cameras were used to monitor and for positioning the meta-aperture to make sure that all of the transmission spectra (including the DC transmission through a slit-only structure that is for normalization) were measured at the same place with respect to the slightly focused incident beam (hence experiencing the same intensity distribution).

## Additional information

**How to cite this article:** Du, L. *et al.* Broadband chirality-coded meta-aperture for photon-spin resolving. *Nat. Commun.* 6:10051 doi: 10.1038/ncomms10051 (2015).

## Supplementary Material

Supplementary Information Supplementary Figures 1-5 and Supplementary Notes 1-2.
