# Supplementary material for: Broadband chirality-coded meta-aperture for photon-spin resolving
Source: Nat Commun. 2015 Dec 2;6:10051. doi: 10.1038/ncomms10051 (PMC4686760; doi:10.1038/ncomms10051)
Supplement: Supplementary Information — Supplementary Figures 1-5 and Supplementary Notes 1-2. [file ncomms10051-s1.pdf]

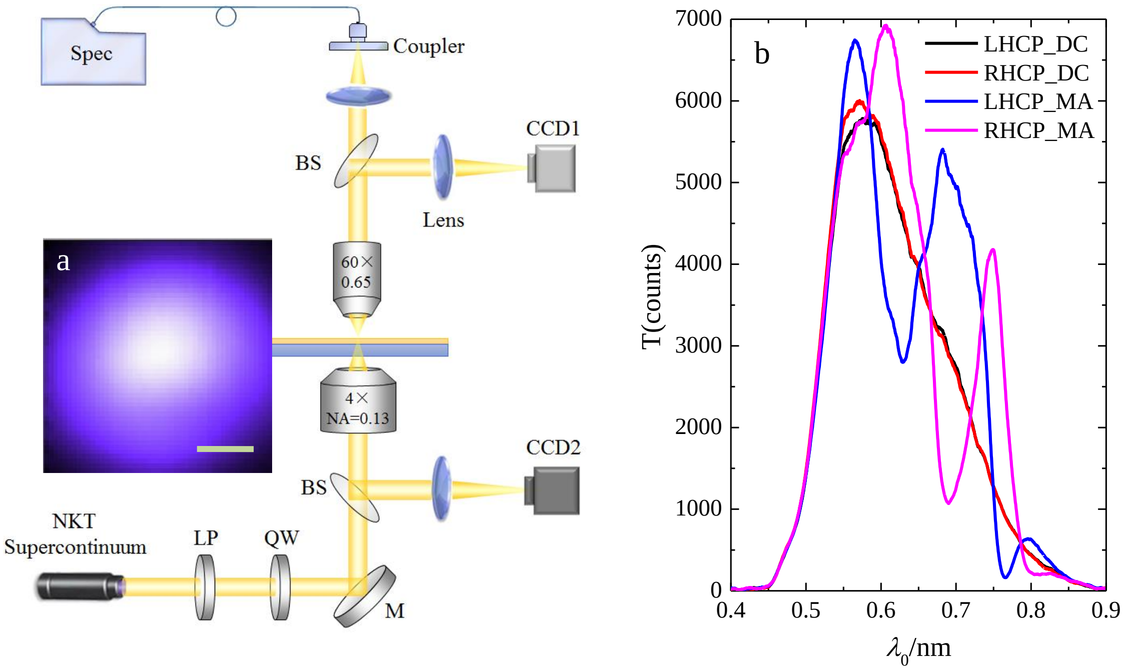

Supplementary Figure 1. Experimental setup. (LP: linear polarizer; QW: 1/4λ waveplate; M: mirror; BS: beam splitter; Spec: spectrometer; CCD: charge-coupled device). A collimated supercontinuum light source (NKT, SuperK Compact) is illuminated normally on to the meta-aperture via a low numerical aperture (NA) objective lens (Olympus, 4×, NA=0.13). A combination of linear polarizer and quarter waveplate was employed to shift the incident polarization between left-handed and right-handed circular polarization. The transmitted light through the meta-aperture is collected with another objective lens (60×, NA=0.7), and is subsequently directed respectively to a CCD camera and a spectrometer with a cubic beam splitter. The CCD cameras were used to monitor and for positioning the meta-aperture to make sure that all of the transmission spectra (including the DC transmission through a slit-only structure that is for the normalization) were measured at the same place with respect to the slightly-focused incident beam (hence experiencing the same intensity distribution). Inset (a) shows the near-field intensity mapping of the focused laser beam impinging onto the sample. The focal spot size is carefully controlled such that the structure is well within the full-width half-maximum (FWHM) area. Inset (b) shows the raw optical transmission spectra through an aperture-only structure (termed as "_DC") and a specific meta_aperture (as "_MA"). The transmission spectra in the main text are obtained by normalizing the transmittance from the meta_aperture with that from the central aperture only (as "*_MA/*_DC").

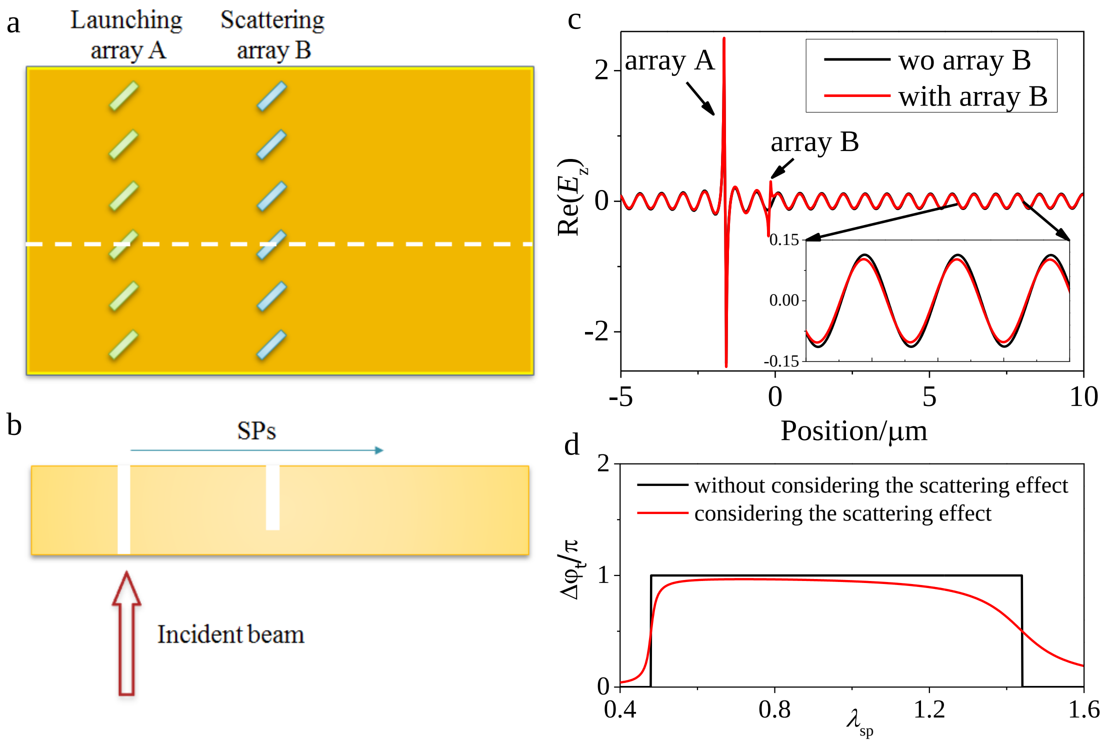

Supplementary Figure 2. **The scattering effect of SPs when encountering an antenna array on the phase difference of SPs excited from the two spin states of CPL.** (a) A designed configuration modeling the scattering effect of SPs, with the cross-sectional view along the white dashed line shown in (b). The configuration includes two antenna columns with the left-side antennas penetrated through the metal film. A laser beam is illuminated onto the structure from the bottom side such that SP wave is launched merely from the left-side antennas ("launching array") and interacts with the right-side antennas ("scattering array") during its propagation. By comparing the energy of SPs with/without the scattering array, we can easily obtain the scattering loss of SPs. (c) The near-field distribution of SPs launched from array A, with and without the scattering array. The inset is a zoom-in view of the curves within 6-8μm, indicating a transmission efficiency of 90% (ξ=0.9) of SP when passing through the scattering array. (d) The phase difference of SPs excited from the two spin states of CPL, considering and without considering the scattering effect, respectively.

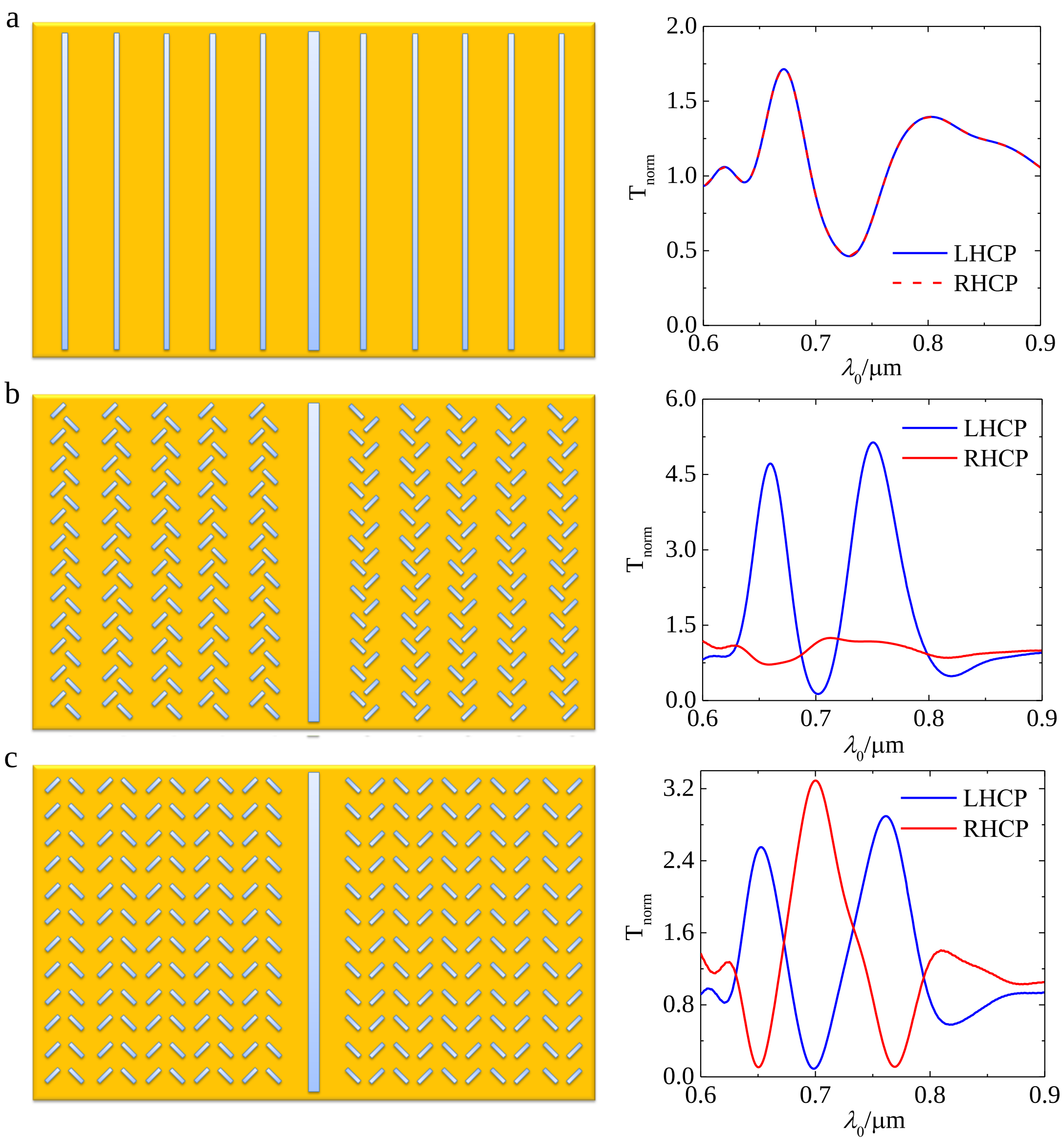

Supplementary Figure 3. **Optical transmission spectra of the same aperture with different decoration structures, for the purpose of comparison.** (a) An ordinary EOT configuration in which the central aperture is surrounded with periodic straight grooves. The symmetry of the structure leads to a spin-degenerated optical transmission. (b) The same aperture decorated with a pair of meta-surfaces that exhibit spin-dependent unidirectional SPPs emission. Such kind of intensity modulation of SPPs could yield, to some extent, helicity-variant optical transmissions, but not in an inverse style. (c) The meta-aperture demonstrated in this article and its corresponding transmission spectra.

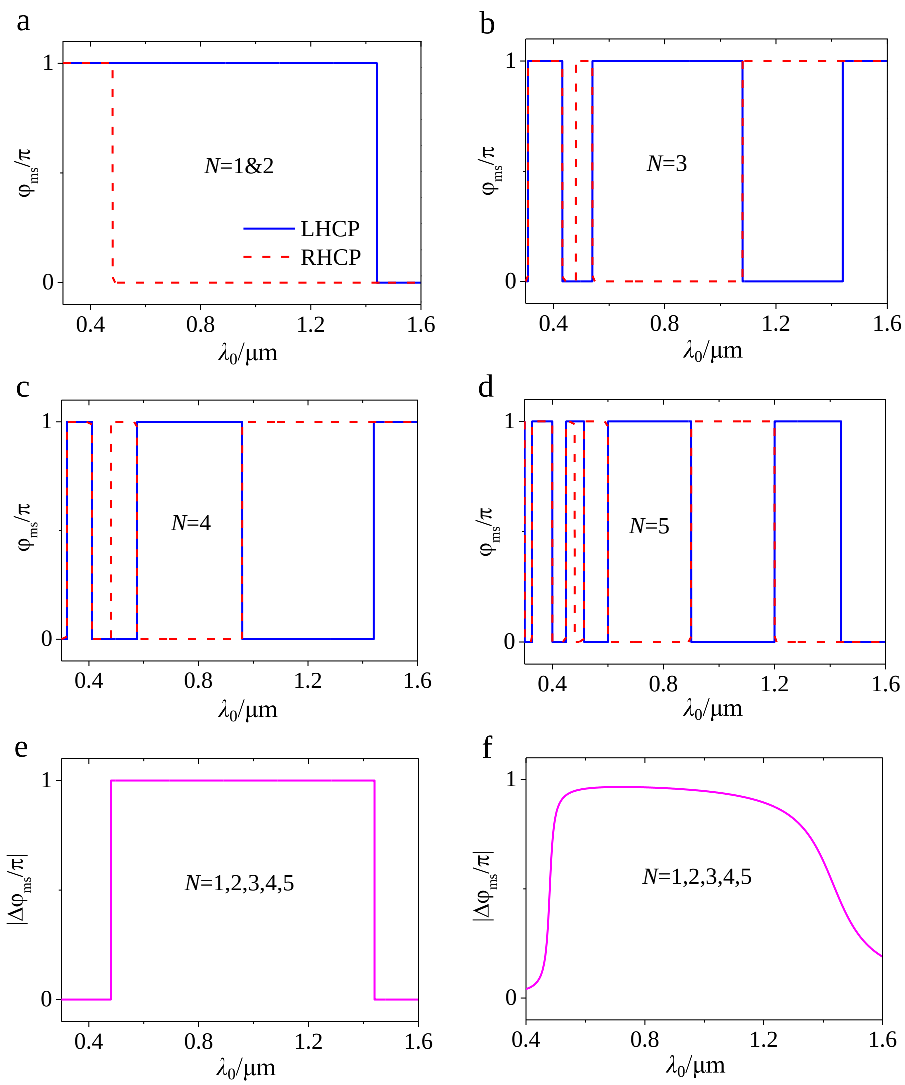

Supplementary Figure 4. **The effect of increasing the number of metasurface seed on the phase of SPs.** (a) - (d) illustrate the phases of SPs induced by the CPL when the number of seed is 1, 2, 3, 4 and 5, respectively, without considering the scattering effect of SPs (assume ξ=1). (e) The differential phase between SPs from left-handed and right-handed CPL without considering the scattering effect. (a) - (d) clearly indicate that the process of increasing an additional metasurface seed with the approach aforementioned maintains the differential phases between SP waves from left-handed and right-handed CPL, although the properties of BP for each of the CPLs are altered. (f) The differential phase between SPs from left-handed and right-handed CPL after considering the scattering effect (assume ξ=0.9 as obtained in Supplementary Figure 2c). The curve of differential phases of SPs keeps unchanged as well when increasing the number of metasurface seed in the case of considering the scattering effect.

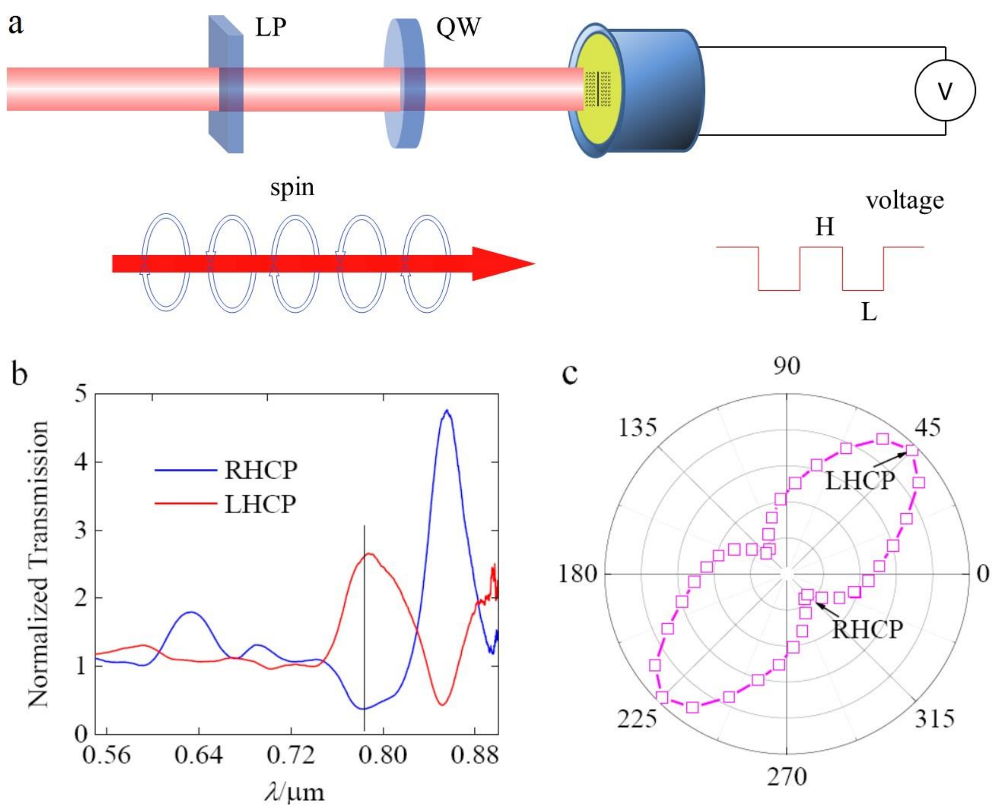

Supplementary Figure 5. Meta-aperture enabled spin-photodiode. (a) Working diagram of the spin-photodiode. The meta-aperture is coated on the window of a compact and low-cost photodiode. By illuminating CPLs onto the photodiode, the spin information of incident photons is transferred to the binary voltage signals, which can readily be integrated with other electronic elements. (b) Transmission spectra of a designed meta-aperture that works at the wavelength of 785 nm. (c) Polarization measurement from a spin-photodiode coated with the designed meta-aperture at 785 nm wavelength. The maximum and minimum values of the measured photocurrent correspond to left-and right-handed polarization states, respectively, validating the effectiveness of the photodiode for distinguishing photon spin states. LP: linear polarizer; QW: quarter waveplate.

Supplementary Note 1: Analytical model of SPs field launched by a metasurface seed

For each of the rectangular nanoantennas composing the metasurface seed as shown in Fig. 3a in the main text, it can approximately be regarded as an in-plane dipolar plasmon source provided the aspect ratio of the structure is sufficiently larger. Assuming that an antenna is φ_{dp}-angled with respect to the *x*-axis (as illustrated in the inset in Fig. 3a in the main text) and illuminated with a circularly polarized light, the electric field of SPs emerging from the antenna can be calculated under the dipole approximation:

$$E_z^j(x,y)=\eta E_0 e^{i\sigma_{\mathrm{s}}\cdot\varphi_{\mathrm{dp}}}\cdot\cos(\varphi_{\mathrm{dp}}-\varphi_j)\frac{1}{\sqrt{r_j}}e^{i(\beta r_j+\varphi_{\mathrm{c}})} \tag{1}$$

where r_j denotes the distance apart from the geometric center of the antenna and φ_j the polar angle with respect to the *x*-axis, respectively. The combined field from a vertical array (assuming it extends to infinity) is simply the summation of the electric field from all of the antennas

$$E_{z,\mathrm{array}}(x,y)=\sum_{j=-\infty}^{\infty}E_z^j=\eta E_0 e^{i\varphi_{\mathrm{c}}}\cdot e^{i\sigma_{\mathrm{s}}\cdot\varphi_{\mathrm{dp}}}\cdot\sum_{j=-\infty}^{\infty}\cos(\varphi_{\mathrm{dp}}-\varphi_j)\frac{1}{\sqrt{r_j}}e^{i\beta r_j} \tag{2}$$

Under the stationery phase approximation:

$$I(\lambda)=\int_a^b f(t)e^{i\lambda g(t)}dt\sim f(c)e^{i\lambda g(c)}\sqrt{\frac{2\pi}{\lambda|g''(c)|}}e^{i\mu\frac{\pi}{4}},\lambda\to\infty \tag{3}$$

Eq. (3) evolves to an analytical one:

$$E_{z,\mathrm{array}}(x,y)\approx\eta E_0\frac{\sqrt{\lambda_{\mathrm{sp}}}}{t_y}e^{i\varphi_{\mathrm{c}}}\cdot\cos(\varphi_{\mathrm{dp}})\cdot e^{i\sigma_{\mathrm{s}}\cdot\varphi_{\mathrm{dp}}}\cdot e^{i\frac{\pi}{4}}\cdot e^{i\beta x} \tag{4}$$

Eq. (4) illustrates an SP plane wave with spin- and orientation-dependent phase. As a result, the total electric field of SPs from the metasurface seed is the superposition of a pair of plane waves:

$$\begin{aligned}E_z(x,y)&=\eta E_0\frac{\sqrt{\lambda_{\mathrm{sp}}}}{t_y}e^{i\varphi_{\mathrm{c}}}\cdot e^{i\frac{\pi}{4}}\cdot[\cos(\varphi_{\mathrm{dp}})\cdot e^{i\sigma_{\mathrm{s}}\cdot\varphi_{\mathrm{dp}}}\cdot e^{i\beta(x-t/2)}+\cos(\varphi_{\mathrm{dp}}+\frac{\pi}{2})\cdot e^{i\sigma_{\mathrm{s}}\cdot(\varphi_{\mathrm{dp}}+\frac{\pi}{2})}\cdot e^{i\beta(x+t/2)}]\\&=\eta E_0\frac{\sqrt{\lambda_{\mathrm{sp}}}}{t_y}e^{i\varphi_{\mathrm{c}}}\cdot e^{i\sigma_{\mathrm{s}}\cdot\varphi_{\mathrm{dp}}}\cdot e^{i\frac{\pi}{4}}\cdot e^{i\beta x}[\cos(\varphi_{\mathrm{dp}})\cdot e^{-i\beta t/2}-\sigma_{\mathrm{s}}\cdot i\sin(\varphi_{\mathrm{dp}})\cdot e^{i\beta t/2}]\end{aligned} \tag{5}$$

In the calculation, we do not consider the scattering effect of SPs when encountering the adjacent antenna array during its propagation. Such a treatment could give us a clearer picture of the physics behind the spin-resolved EOT while does not lose much the precision. Under two special cases, i.e., φ_{dp} = +π/4 and –π/4, Eq. (5) becomes:

$$E_{z,\wedge}(x,y)==\frac{1}{t_y}\cdot\sqrt{\frac{\pi}{\beta}}\cdot\eta\cdot E_0\cdot e^{i\varphi_{\mathrm{c}}}\cdot e^{i\pi/4}\cdot e^{i\beta x}[e^{-i(\beta t/2-\sigma_{\mathrm{s}}\cdot\pi/4)}+e^{i(\beta t/2-\sigma_{\mathrm{s}}\cdot\pi/4)}] \tag{6}$$

and

$$E_{z,\vee}(x,y)==\frac{1}{t_y}\cdot\sqrt{\frac{\pi}{\beta}}\cdot\eta\cdot E_0\cdot e^{i\varphi_{\mathrm{c}}}\cdot e^{i\pi/4}\cdot e^{i\beta x}[e^{-i(\beta t/2+\sigma_{\mathrm{s}}\cdot\pi/4)}+e^{i(\beta t/2+\sigma_{\mathrm{s}}\cdot\pi/4)}] \tag{7}$$

Here, we use the symbols of "∧" and "∨"to mimic the appearance of the metasurface seed when φ_{dp} = +π/4 and –π/4, respectively.

Supplementary Note 2: Analytical model of the phase of SPs when increasing the number of metasurface seeds

As discussed in the main text, the basic principle of the process to improve the extinction ratio is that the out-of-phase feature of the structure should not be modified when increasing the number of metasurface seeds. For the metasurface as illustrated in Fig. 4a in the main text, it is interpreted as an assembly of multiple seeds with alternate orientation and 2*t* increment of the separation. For simplicity, it is termed as MS=[S1,S2,S3,S4,S5]=[(*t*, ∧),(3*t*, ∨),(5*t*, ∧),(7*t*, ∨),(9*t*, ∧)]. As all of the metasurface seeds share the geometric center, the consolidated phase of the entire structure is expressed as:

$$\Phi^{N}_{\mathrm{ms},\sigma_{\mathrm{s}}} = \sum_{i}^{N} \mathrm{BP}_{\sigma_{\mathrm{s}}}(t_i, \varphi_{\mathrm{dp},i}) \qquad \sigma_{+} = \mathrm{LHCP}, \quad \sigma_{-} = \mathrm{RHCP}, \quad \varphi_{\mathrm{dp},i} = \pm\frac{\pi}{4} \tag{8}$$

where $\mathrm{BP}_{\sigma\mathrm{s}}$ represents the binary phase induced by a metasurface seed.

Substituting the expression for $\mathrm{BP}_{\sigma\mathrm{s}}$ as derived in Supplementary Note 1, we can obtain:

$$\sum_{i}^{N} \mathrm{BP}_{\sigma_{\mathrm{s}}}(t_i, \varphi_{\mathrm{dp},i}) = \cos(\frac{\beta t}{2} - \sigma_{\mathrm{s}} \cdot \frac{\pi}{4}) + \cos(\frac{3}{2}\beta t + \sigma_{\mathrm{s}} \cdot \frac{\pi}{4}) + \ldots + \cos(\frac{2N-1}{2}\beta t + (-1)^N \sigma_{\mathrm{s}} \cdot \frac{\pi}{4}) \tag{9}$$

In case the metasurface composes of odd number of seeds, Eq. (9) will be evolved to:

$$\begin{aligned}
\Phi^{N}_{\mathrm{ms},\sigma_{\mathrm{s}}} &= \cos(\frac{\beta t}{2} - \sigma_{\mathrm{s}} \cdot \frac{\pi}{4}) + [\cos(\frac{3}{2}\beta t + \sigma_{\mathrm{s}} \cdot \frac{\pi}{4}) + \cos(\frac{5}{2}\beta t - \sigma_{\mathrm{s}} \cdot \frac{\pi}{4})] + \ldots \\
&+ [\cos(\frac{2N-3}{2}\beta t + \sigma_{\mathrm{s}} \cdot \frac{\pi}{4}) + \cos(\frac{2N-1}{2}\beta t - \sigma_{\mathrm{s}} \cdot \frac{\pi}{4})] \\
&= \cos(\frac{\beta t}{2} - \sigma_{\mathrm{s}} \cdot \frac{\pi}{4}) + 2\cos(2\beta t)\cos(\frac{\beta t}{2} - \sigma_{\mathrm{s}} \cdot \frac{\pi}{4}) + \ldots + 2\cos[(N-1)\beta t]\cos(\frac{\beta t}{2} - \sigma_{\mathrm{s}} \cdot \frac{\pi}{4}) \\
&= [1 + 2\sum_{i=1}^{n} \cos(2i\beta t)]\cos(\frac{\beta t}{2} - \sigma_{\mathrm{s}} \cdot \frac{\pi}{4}) \qquad n = \frac{N-1}{2}
\end{aligned} \tag{10}$$

While the formula becomes to the following expression when N is even:

$$\begin{aligned}
\Phi^{N}_{\mathrm{ms},\sigma_{\mathrm{s}}} &= [\cos(\frac{\beta t}{2} - \sigma_{\mathrm{s}} \cdot \frac{\pi}{4}) + \cos(\frac{3}{2}\beta t + \sigma_{\mathrm{s}} \cdot \frac{\pi}{4})] + \cos(\frac{5}{2}\beta t - \sigma_{\mathrm{s}} \cdot \frac{\pi}{4}) + \cos(\frac{7}{2}\beta t + \sigma_{\mathrm{s}} \cdot \frac{\pi}{4})] + \ldots \\
&+ [\cos(\frac{2N-3}{2}\beta t - \sigma_{\mathrm{s}} \cdot \frac{\pi}{4}) + \cos(\frac{2N-1}{2}\beta t + \sigma_{\mathrm{s}} \cdot \frac{\pi}{4})] \\
&= 2\cos(\beta t)\cos(\frac{\beta t}{2} + \sigma_{\mathrm{s}} \cdot \frac{\pi}{4}) + 2\cos(3\beta t)\cos(\frac{\beta t}{2} + \sigma_{\mathrm{s}} \cdot \frac{\pi}{4}) + \ldots + 2\cos[(N-1)\beta t]\cos(\frac{\beta t}{2} + \sigma_{\mathrm{s}} \cdot \frac{\pi}{4}) \\
&= 2\sum_{i=1}^{n} \cos[(2i-1)\beta t]\cos(\frac{\beta t}{2} + \sigma_{\mathrm{s}} \cdot \frac{\pi}{4}), \qquad n = \frac{N}{2}
\end{aligned} \tag{11}$$

Finally, the out-of-phase/in-phase feature of the metasurface responding to the spin pair of CPL can be determined by multiplying the consolidated phases:

$$\Phi^{N}_{\mathrm{ms},\sigma_{-}} \cdot \Phi^{N}_{\mathrm{ms},\sigma_{+}} = \gamma^2 \cos(\frac{\beta t}{2} - \frac{\pi}{4})\cos(\frac{\beta t}{2} + \frac{\pi}{4}) = \gamma^2 \Phi^{1}_{\mathrm{ms},\sigma_{-}} \cdot \Phi^{1}_{\mathrm{ms},\sigma_{+}} \tag{12}$$

where

$$\gamma = \begin{cases} 2\sum_{i=1}^{n}\cos[(2i-1)\beta t], & n = \frac{N}{2}, \quad N = 2,4,6,... \\ 1+2\sum_{i=1}^{n}\cos(2i\beta t), & n = \frac{N-1}{2}, \quad N = 3,5,7,... \end{cases} \tag{13}$$

Eq. (12) clearly indicates that the process of increasing an additional metasurface seed with the approach aforementioned maintains the relative phases between SP waves from left-handed and right-handed CPL (Supplementary Figure 4e), although the properties of binary phase for each of the circular polarizations are altered with the effect from γ (Supplementary Figure 4a-d). Further calculation indicates that, after considering the scattering effect of SP during its propagation, the curve of differential phase when increasing the number of metasurface seed keeps unchanged as well, as illustrated in Supplementary Figure 4f.
